# *Trolliusaustrosibiricus* (Ranunculaceae), a new species from South Siberia

**DOI:** 10.3897/phytokeys.115.30863

**Published:** 2019-01-28

**Authors:** Andrey Erst, Alexander Luferov, Victoria Troshkina, Dmitry Shaulo, Alexander Kuznetsov, Kunli Xiang, Wei Wang

**Affiliations:** 1 Laboratory Herbarium (NS), Central Siberian Botanical Garden, Russian Academy of Sciences, Zolotodolinskaya st. 101, 630090, Novosibirsk, Russia Central Siberian Botanical Garden, Russian Academy of Sciences Novosibirsk Russia; 2 Laboratory Herbarium (TK), Tomsk State University, Lenin st. 36, 634050, Tomsk, Russia Tomsk State University Tomsk Russia; 3 I.M. Sechenov First Moscow State Medical University, Izmailovsky Boulevard, 8, 105043, Moscow, Russia I.M. Sechenov First Moscow State Medical University Moscow Russia; 4 State Key Laboratory of Systematic and Evolutionary Botany, Institute of Botany, Chinese Academy of Sciences, 100093, Beijing, China Institute of Botany, Chinese Academy of Sciences Beijing China; 5 University of Chinese Academy of Sciences, 19 Yuquan Road, Beijing 100049, China University of Chinese Academy of Sciences Beijing China

**Keywords:** Ranunculaceae, *
Trollius
*, new species, South Siberia, Russia

## Abstract

*Trolliusaustrosibiricus* Erst & Luferov, **sp. nov.**, a new species from Russian South Siberia is described and illustrated. This new species is endemic to Western and Central Siberia. Morphologically, it is close to the East Asian species *T.chinensis* and *T.macropetalus*. However, it differs from the aforementioned species due to the morphology of the rhizomes, aerial shoots, sepals and petals. This species is also distinguished from *T.asiaticus*, which is widespread in Russia (Western and Eastern Siberia), Mongolia, China, north-eastern Kazakhstan and in the northeast of the European part of Russia, in having a smaller number of sepals, longer persistent styles and petals longer than sepals. In addition, an identification key for all Russian species is given and all species have been discussed.

## Introduction

*Trollius* L. (Ranunculaceae) is distributed in temperate to arctic regions of the Northern Hemisphere and has two centres of diversity in SW China and the area ranging from Siberia, the Pamirs and Kashmir (Doroczewska 1974). The genus is characterised by conspicuous orange- or yellow-coloured flowers, similarly coloured petals, subscapose habit and ternate or deeply 3-lobed leaves (Kadota 1987). *Trollius* is distinguished by an unusual floral structure with petals divided into blade, pit (nectarostigma) and claw (Wang et al. 2010). Length ratio of nectaries to stamens has been considered to be one of the most important morphological characters for species delimitation (Schipczinsky 1937, Siplivinsky 1972, Kadota 1987, Tamura 1995, Luferov et al. 2018). Species belonging to the T.sect.Longipetala Dorosz. are characterised by linear, flat, thin petals, which are longer than the sepals or nearly equal to them. All representatives belonging to this group, except *T.asiaticus*, are common to the Far East part of Asia. All Siberian species are characterised by petals shorter or equal to sepals. When carrying out a revision of the genus for Russia, we focused on specimens whose petals are much longer than the sepals. Further revision of the herbarium material allowed us to describe a new species of *Trollius* from South Siberia.

## Methods

**Figure 1.**
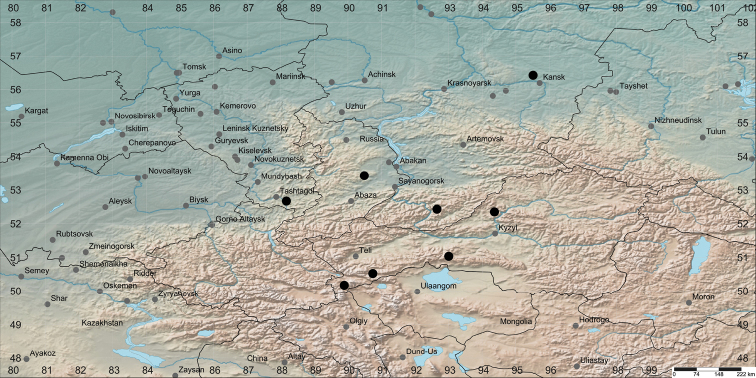
Distribution map of *Trolliusaustrosibiricus*.

The revision of herbarium material was undertaken in the herbaria LE, MHA, ALTB, NS and NSK (Thiers 2017). The drawings of *Trolliusaustrosibiricus* are based on the images of the type specimens (NS-0013097). The photographs in the field were taken by a Nikon D90 camera. The morphological characters were measured using AxioVision 4.8. The flowering and fruiting periods and habitats are given as cited on the collector’s labels. The IUCN Red List Categories and Criteria (IUCN 2016) were applied to assess the conservation status. All revised localities of *Trolliusaustrosibiricus* mentioned in the paper are shown on a map (Fig. 1) made with SimpleMappr (http://www.simplemappr.net).

## Taxonomy

### Trollius austrosibiricus

Taxon classificationPlantaeRanunculalesRanunculaceae

Erst & Luferov sp. nov.

urn:lsid:ipni.org:names:77194177-1

Figs 2 , 3

#### Type.

RUSSIA. Tuva Republic: Mongun-Tayga region, Tsagan-Shibetu ridge, the upper Barlyk river, valley of the right tributary, the lower part of the south-eastern slope (5º), forb-grass-sedge steppe meadow, 2350 m alt., flowering, 22 Jul 1980, *V. Khanminchun, M. Danilov & P. Enns* without collector number (holotype: NS barcode 0013097!).

#### Diagnosis.

*Trolliusaustrosibiricus* is morphologically close to *T.chinensis* Bunge and *T.macropetalus* (Regel) Fr.Schmidt. It differs from these species in simple rhizomes, shorter aerial shoots, smaller flowers and shorter persistent styles. The new species is distinguished from *T.asiaticus* L. by a smaller number of sepals, longer persistent styles and petals longer than sepals.

#### Description.

*Herbs* perennial, (20–)40–70 cm high. *Rhizomes* simple or slightly branching, short, erect or arched, with a bundle of adventitious roots. *Stems* straight, simple, less often weakly branched, slightly grooved. *Basal leaves* (1–)2–4, spirally-alternate, congested in a basal rosette; petioles 10–25(–35) cm long; blades 4–7 × 5–8 cm, rhomboid, 3–5(–7)-lobed, segments dissected almost to the midrib into lobes, ending sharply with edges dentate. *Cauline leaves* 2–5(–7), opposite, with short petioles or sessile, gradually smaller towards the apex. *Inflorescence* terminal, 1(–2)-flowered. *Flowers* 3.5–4.5(–5.5) cm diam.; pedicels 5–12 cm long, elongating in fruit up to 8–15 cm long; sepals 8–10(–14), 1.3–2.4 × 0.8–1.5 cm, rhombic-ovate or broadly elliptic, reddish-orange or yellow-orange; petals 9–18, 2–2.8 × 0.2–0.3 cm, oblong-lanceolate, slightly wider at middle, base narrow cuneate, apex acute, orange- or reddish-orange coloured, nectarostigma 2.5–3 mm from base; stamens more than (9)10, filaments 7-11mm long, anthers 1.5–2(–2.5) mm long, linear; *Fruits* aggregate, with 9–14(17) follicles, 10–15 mm long, persistent style 1.5–3.0 mm long, slightly incurved.

**Figure 2.**
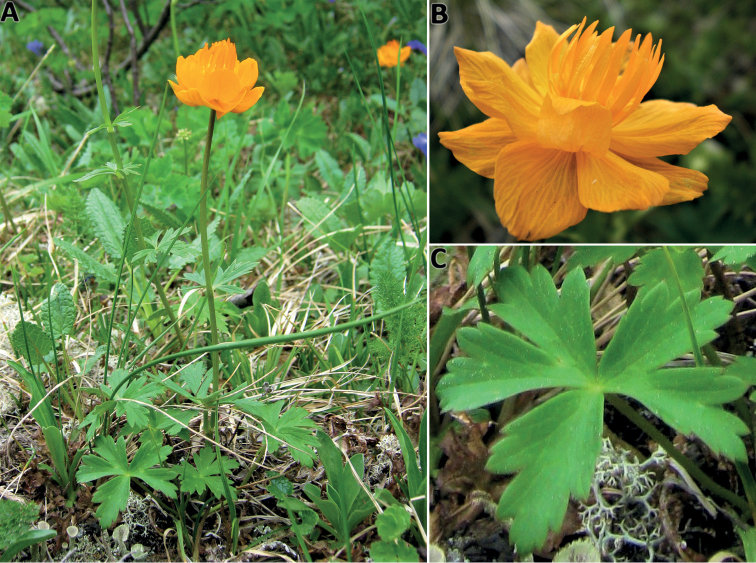
Photograph of *Trolliusaustrosibiricus*. **A** Flowering plant **B** Flower **C** Leaf laminae (Photographs by E. Balde and A. Erst).

#### Specimens seen

**(paratypes). Russia. Tuva Republic**: Western Sayan, Kurtushibinskiy ridge, the upper Mynas river, right tributary of the Hut river, forb-grass meadow, 1080 m alt., 6 Jul 1989, D. Shaulo & I. Kovaleva 4695 (NS barcode 0013098!); Mongun-Tayga region, valley of Tolayty river, yernik-sedge wetland meadow, 2500 m alt., 26 Jul 1980, M. Danilov & A. Krytsin 932 (NS barcode 0013096!); the northern slope of the East Tannu-Ola ridge, the average flow of Elegest river, floodplain, No., 22 Jul 1973, V. Khanminchun & V. Dyukov 2270 (NS barcode 0013095!); **Kemerovo Oblast**: Tashtagol region, the village of Ust-Kabyrza 4 km above Pysas river, 52°48’N, 88°28’E, 450 m alt., grass meadow, haymaking, 11 Sep 2000, I. M. Krasnoborov, A. I. Shmakov, D. Germann, S. Kostyukov, E. Antonuk, P. Kosachev & A. Vashchenko 207 (NS barcode 0013094!); **Krasnoyarsk Krai**: Abansky region, near Ustyanskoye village, the upper part of the eastern slope, upland meadow, 6 Jul 1956 T. Vagina (NS barcode 0013093!); Ermakovsky district, valley of the US river, elevation 852 m alt., 52.2847°N, 93.2517°E, 03 Jul 2010, I.V. Khan & E.A. Balde 152 (NSK barcode 0028601!); **Khakassia Republic**: Abansky ridge, near Biskamzha station, southern slope, burning, 53.25N, 089.30E, 700 m alt., 4 Jun 1991, E. Ankipovich (NS barcode 0013092!).

#### Affinities.

*Trolliusaustrosibiricus* is morphologically close to *T.chinensis* Bunge and *T.macropetalus* (Regel) Fr.Schmidt. It is well distinguished by simple rhizomes (rather than by the multi-headed basal part of the plant, as in *T.chinensis* and *T.macropetalus*), shorter aerial shoots, smaller flowers and shorter persistent styles (Table 1). *Trolliuschinensis* is an East Asian species occurring in Russia (Primorsky and Khabarovsk territories, Sakhalin), in the north and northeast of China and on the Korean peninsula (Schipczinsky 1937, Siplivinsky 1972, Doroczewska 1974, Kitagawa 1979, Woroshilov 1982, Luferov 1991, 2004).

This new species is distinguished from *T.asiaticus* L. by a smaller number of sepals, longer persistent styles and petals longer than sepals (Table 1). *Trolliusasiaticus* grows mainly in extra-tropical Asia (Western and Eastern Siberia, Mongolia, northeast Kazakhstan and China), as well as in the northeast of European Russia (Schipczinsky 1937, Siplivinsky 1972, Doroczewska 1974, Borodina-Grabovskaya 2001, Friesen 2003).

**Figure 3.**
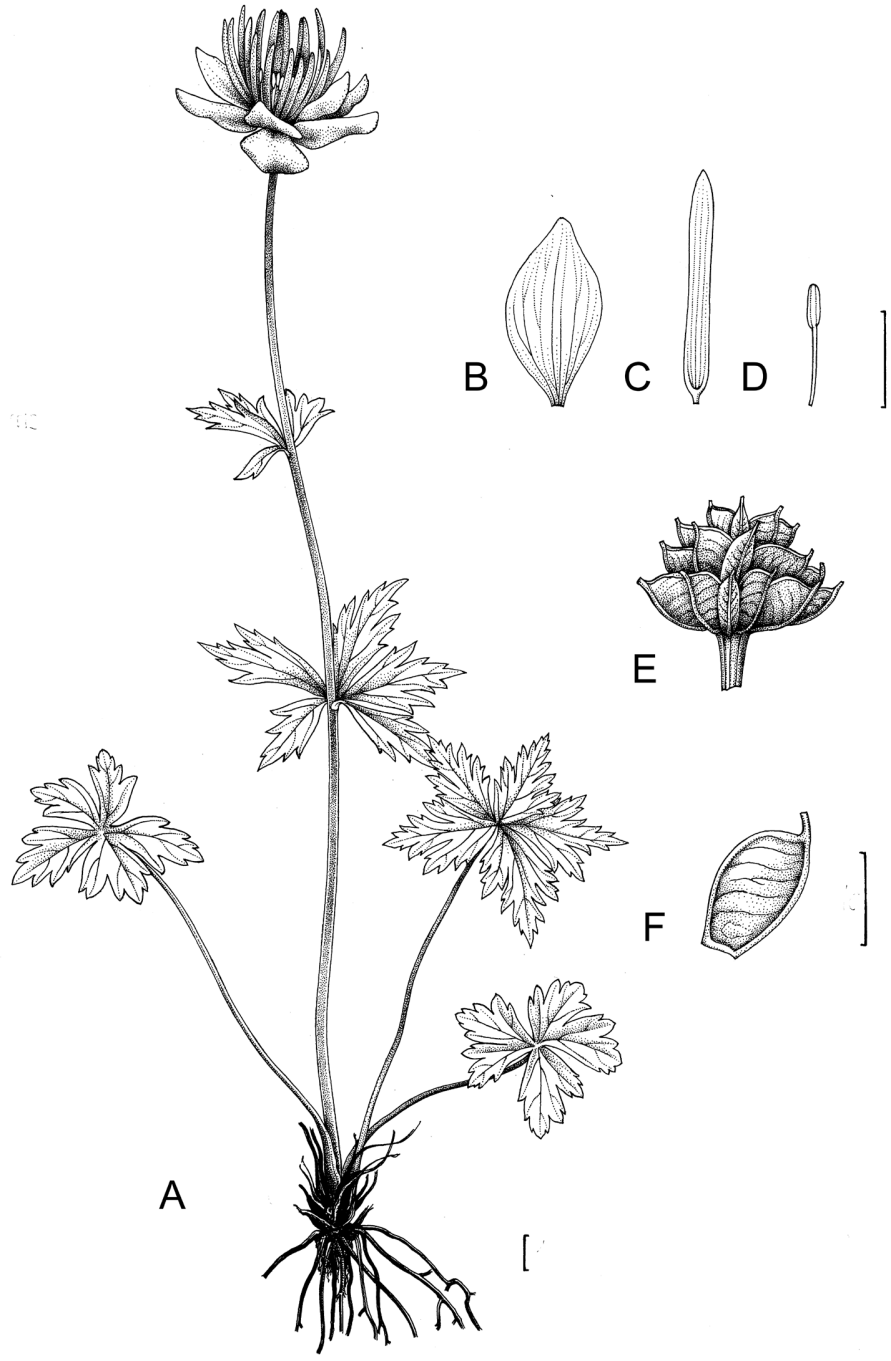
*Trolliusaustrosibiricus*. **A** General view **B** Sepal **C** Petal **D** Stamen **E** Fruit **F** Follicle. Scale bar: 1 cm (**A–F**).

**Table 1.** Morphological comparison between *Trolliusaustrosibiricus* and related species.

Characters	* T. austrosibiricus *	* T. chinensis *	* T. macropetalus *	* T. asiaticus *
**Stem height, cm**	(20)40–70	70–150 (180)	70–150 (180)	20–75
**Underground organs**	simple rhizomes	multi-headed basal part	multi-headed basal part	multi-headed basal part
**Length/width of the basal leaf blade**	4–7/5–8	6–15/7–25	6–15/7–25	4–7/5–8
**Number of flowers on shoot**	1(2)	2–7(1)	2–7(1)	1–2(3)
**Flower diameter, cm**	3.5–4.5(5.5)	4–5(6)	(4)5–7(8)	3–4(5)
**Number of sepals**	8–10(14)	8–12	5–7	10–20
**Petal length, mm**	20–28	25–35	30–35 (40)	14–22
**Petal apex**	Acute, narrowed smoothly from the middle part of the petal	Acute, narrowed abruptly upwards	Acuminate, narrowed smoothly upwards	Rounded
**Distance from nectary pit to petal base, mm**	2.5–3	3.5–4	3.5–4.5	1.5–2
**Sepals/Petals length ratio**	<1	<1	<1	≥1
**Petal/Stamen length ratio**	2–3.5/1	2–3/1	3.5–5/1	1.5–2/1
**Follicle length, mm**	10–15	14–18	14–22	8–12
**Style length, mm**	1.5–3	3–4	3.5–5	0.5–1(2)
**Persistent style shape**	Almost erect at the base, above: bent arc-like inwards	Bent outwards at the base, above: bent slightly arc-like towards the centre of the flower	Bent outwards at the base, above: bent slightly arc-like towards the centre of the flower	Bent sharply arc-like towards the centre of the flower

#### Phenology.

Flowering from April–early May; fruiting in May.

#### Distribution.

*Trolliusaustrosibiricus* is endemic to mountainous areas of the southern part of Western and Central Siberia. Russia: Tuva Republic, Kemerovo district, Krasnoyarsk territory, Khakassia (Figure 1).

#### Habitat and ecology.

*Trolliusaustrosibiricus* grows in subalpine and forest zones, in moist valleys at 350–2400 m elevation. It occurs in forest glades and fringes, in mixed-grass and mixed-grass-cereal dry and swampy meadows, along the banks of rivers, streams and small ponds with fresh water.

#### Etymology.

The specific epithet of the new species is derived from the type locality, South Siberia, Russia.

#### Preliminary conservation assessment.

No appropriate data on abundance and/or distribution of the taxon are available. It can be included in the Not Evaluated (NE) category of IUCN Red List Categories (IUCN 2016) as it lacks adequate information to make a direct or indirect assessment of its risk of extinction based on its distribution and/or population status.

## Discussion

According to our data, 16 species of *Trollius* occur in Russia. The “Flora of the USSR” includes 11 species of *Trollius*, one of which is not found in Russia: *T.dschungaricus* Regel is confined to the east of Central Asia and China (Schipczinsky 1937). The greatest species diversity is observed in Siberia (12 species: our data); 11 species of *Trollius* (Friesen 2003) were previously indicated for this region, of which we recognise 9 and *T.sayanensis* (Malyschev) Sipl. and *T.vicarius* Sipl. are synonyms to *T.asiaticus* L and *T.uniflorus* Sipliv. Nine species grow in the Far East (Luferov et al. 2018), 5 species of *Trollius* are found in the European part of Russia (Tzvelev 2001) and 1 species occurs in the Caucasus (Borodina-Grabovskaya 2012). Many species that have been described recently from Russia require more detailed investigation and evidence of species independence (Luferov et al. 2018). Many taxa of the genus have intermediate characteristics of the groups described by A. Doroczewska (1974) as sections. The groups considered in this monograph require verification, since many species exhibit intermediate features between sections. Molecular phylogeny has not yet been developed, but there are some works related to a small number of species that do not consider the classification of groups (Despres et al. 2003, Wang et al. 2010). Representatives of the Trollius section occur in Russia (petals 6–14 mm long, linear, apex rounded or spatulate, with no groove at the base; plants of Asia, Europe and the Caucasus): *T.europaeus*, *T.altaicus* and *T.ranunculinus*; the *Longipetala* sections (petals 7–40 mm long, oblong, narrow, flat, thin, not thickened and dull, similar to sepals and usually orange): *T.macropetalus*, *T.ledebourii*, *T.austrosibiricus*, *T.asiaticus*, *T.kytmanovii* and *T.apertus*; the *Insulaetrollius* sections: (petals 2–12 mm long, linear, extended or pyriform, grooved at the base; plants of the Okhotsk-Japan-Kamchatka region): *T.riederianus*, *T.sibiricus*, *T.uniflorus*, *T.japonicus*, *T.miyabei*, *T.membranostylis* and *T.chartosepalus*. It should be noted that many morphological characters used to distinguish between species and groups of species are not fully developed, do not cover the entire morphological diversity of the genus *Trollius* and are very variable (many hybridogenic taxa have not yet been described, many species ecomorphs are not yet known). For proper identification, classification and understanding of groups in the genus *Trollius*, complex studies are needed, including micro- and macro-morphological, molecular phylogenetic, cytogenetic etc. This paper presents the results of taxonomic studies that enabled development of the key to identification of the *Trollius* species, based on the novel morphological and rhythmological features: for example, indication of the falling (in *T.chartosepalus*) and non-falling sepals (in other species), shape, the size of sepals, petals, persistent styles, their size ratio, the colour of the flower elements, leaflets and stigmas.

According the most recent data, the key for *Trollius* identification (from Russia) is provided by us.

### Key to identification species of the genus *Trollius* from Russia

**Table:** 

1	Flowers globular, closed due to overlapping sepal edges; petals, stamens and pistils not visible during flowering	*** T. europaeus ***
–	Flowers bowl-shaped, saucer-shaped or, if globular, always open: sepal edges do not overlap; petals, stamens and pistils visible during flowering	**2**
2	Petals longer than sepals and protrude from the flower	**3**
–	Petals equal to sepals or shorter	**4**
3	Sepals 5–7. Petals 30–40 mm long, 1.5–2 times longer than sepals. Persistent styles 3.5–5 mm long	*** T. macropetalus ***
–	Sepals 8–14. Petals 20–28 mm long, 1.2–1.5 times longer than sepals. Persistent styles 1.5–3 mm long	*** T. austrosibiricus ***
4	Petals 1.5–2 times longer than stamens	**5**
–	Petals shorter	**8**
5	Persistent styles and stigmas purplish-black or often blackening. Persistent styles 2.5–3 mm long (Plants from the Altai Republic)	*** T. altaicus ***
–	Persistent styles and stigmas light green or yellow-green. Persistent styles less than 2 mm long	**6**
6	Plants 80–100 cm high. Sepals 5–9 (12)	*** T. ledebourii ***
–	Plants 20–75 cm high. Sepals 10–20	**7**
7	Sepals reddish-orange. Persistent styles 0.5–1 mm long	*** T. asiaticus ***
–	Sepals yellow-orange or yellow. Persistent styles 1.5–2 mm long	*** T. kytmanovii ***
8	Plants bloom prior to leaf expansion. Sepals white or pale cream, finely dentate along the edge, do not fall long after flowering. Leaflets up to 25 mm long. Persistent styles 8–18 mm long, equal to or longer than the ovary, thin, straight or slightly curved	*** T. chartosepalus ***
–	Plants bloom with leaves developed. Sepals from pale yellow to orange, typically smooth-edged, fall at the end of flowering. Leaflets up to 15 (18) mm long. Persistent styles not more than 5 mm long, several times shorter than the ovary, more or less thickened, incurved	**9**
9	Persistent styles 4–5 mm long, 3 times shorter than leaflets. Sepals golden-yellow	*** T. ranunculinus ***
–	Persistent styles less than 3 mm long, 5–7 times shorter than leaflets	**10**
10	Petals equal to stamens, 1–3 mm longer or shorter	**11**
–	Petals 2–2.5 times shorter than stamens	**15**
11	Sepals 5–7	**12**
–	Sepals 9–12	**14**
12	Petals oblong-obovate, apex cuneate. Persistent styles 0.4–1.2 mm long	*** T. apertus ***
–	Petals obovate or spatulate, apex rounded. Persistent styles not less than 2 mm long	**13**
13	Leaf blades round-pentagonal, 4–8 cm long, 5–10 cm wide, dentate with triangular acute and sharp teeth. Flowers 3–4 cm in diameter. Sepals yellow-orange or yellow. Petals are reddish-orange, equal to stamens or 1–3 mm longer. Leaflets with arcuate, unbreakable persistent styles 2–3 mm long	*** T. riederianus ***
–	Leaf blades are rounded- reniform, 8–14 cm long, 10–24 cm wide, dentate with narrow-triangular acute and sharp teeth. Flowers 2.5–3.5 cm in diameter. Sepals pale yellow or yellow-orange. Petals orange, 1–3 mm shorter than stamens. Leaflets straight or slightly arcuate, with longer (3–4.5 mm long), thin, brittle persistent style	*** T. japonicus ***
14	Plants up to 40 cm high. Stem simple, with 1 flower. Sepals grey-yellow. Petals narrow-linear, acute, yellow-orange, 1–3 mm longer than stamens. Pedicels up to 10 cm long with fruits up to 20 cm. Leaflets are light green. (Plants from Siberia and the mainland of the Far East)	*** T. sibiricus ***
–	Plants 70–120 cm high. Stems branched, with 2–5 flowers, less often simple. Sepals yellow-orange or golden-yellow. Petals obovate or spatulate, blunt, orange, equal to stamens in length. Pedicles 2–5 cm long with fruits up to 10 cm. Leaflets reddish-brown later blackening. (Plants from Sakhalin Island)	*** T. miyabei ***
15	Sepals 5–6. Persistent styles up to 1.4 mm long, subulate, straight or slightly curved	*** T. uniflorus ***
–	Sepals 9–12. Persistent styles about 2 mm long, with flattened edges, webbed, arched	*** T. membranostylis ***

## Supplementary Material

XML Treatment for Trollius austrosibiricus
